# Susceptibility of *Ficus carica* L. Cultivars to Fruit Colonization by *Aspergillus* spp. and Its Relationship with Mycotoxin Contamination in Industrial Batches

**DOI:** 10.3390/foods15132289

**Published:** 2026-06-26

**Authors:** Zakaria Janfi, María Ángeles Romero-Martín, Diego Cabello, Ana Gordon, María Teresa García-López, Juan Moral

**Affiliations:** 1 UCOLIVO Group, EPICON Laboratory, Department of Agronomy, Rabanales Campus, University of Cordoba, 14071 Cordoba, Spain; z92jajaz@uco.es (Z.J.);; 2 ENZOEM, Competitive Research Unit on Zoonoses and Emerging Diseases, Rabanales Campus, University of Cordoba, 14071 Cordoba, Spain; 3 Department of Cell Biology, Physiology, and Immunology, Rabanales Campus, University of Cordoba, 14071 Cordoba, Spain

**Keywords:** *Ficus carica*, aflatoxins, ochratoxin A, *Aspergillus* section *Flavi*, *Aspergillus* section *Nigri*, cultivar susceptibility, ostiole, biocontrol, dried figs, food safety

## Abstract

The common fig (*Ficus carica* L.) is susceptible to mycotoxin contamination, raising food safety concerns given the strict maximum levels for aflatoxins (AF) and ochratoxin A (OTA) established by Regulation (EU) 2023/915 for dried figs. We characterized the susceptibility of three commercially relevant cultivars from the Jerte Valley (‘Calabacita’, ‘Cuello Dama Blanco’, and ‘Granito’) to *Aspergillus* section *Flavi* colonization under controlled and orchard conditions. These findings were integrated with an industry surveillance dataset (2014–2023; 326 batches), with cultivar comparisons restricted to the comparable period 2020–2023 (184 batches), from a local fig-processing cooperative. Under controlled inoculation, ‘Calabacita’ consistently showed the highest internal colonization and proportion of figs colonized, whereas ‘Granito’ showed the lowest values. Sealing the ostiole reduced colonization across cultivars and abolished cultivar differences, thereby supporting the ostiole as the primary entry route. Fruit susceptibility increased with fruit size across all three cultivars. Dry conidial inoculation, emulating natural airborne spread, resulted in substantially higher colonization than inoculation with an aqueous conidial suspension, both in severity (100% vs. 3.7%) and incidence (100% vs. 7.3%). Orchard assays were consistent with the controlled inoculation results: ‘Calabacita’ showed higher section *Flavi* colonization than ‘Cuello Dama Blanco’ and ‘Granito’, which did not differ from each other. Fruit position did not affect colonization by section *Flavi*, but it significantly affected colonization by section *Nigri*, which was higher in dropped fruits across cultivars. AF-OTA co-occurrence was restricted to ‘Granito’ (3 batches; 2.6%) and was absent in ‘Calabacita’. Surveillance data (2020–2023) were consistent with the experimental findings. ‘Calabacita’ showed predominantly AF-related non-compliance (with AFB_1_ and AFTA exceedances reaching 25.7%), whereas ‘Granito’ showed higher OTA exceedance (10.5% vs. 1.4%), reflecting distinct contamination profiles between cultivars. This study informs AF and OTA management strategies in dried figs based on cultivar susceptibility profiling, prescreening systems prior to commercialization, and reduction in fruit–soil contact during drying.

## 1. Introduction

The common fig (*Ficus carica* L.) is a high-value Mediterranean crop that is particularly vulnerable to mycotoxin contamination, posing a critical food safety concern in dried fig production systems [1,2]. Within the European Union (EU), fig cultivation has gained renewed consideration, not only for figs’ economic value but also for their remarkable resilience to climate change. Compared with many other fruit trees, figs require relatively low water inputs, making them particularly well-suited to increasingly arid Mediterranean environments [3]. Spain has consolidated its position as the leading fig producer in the EU, ranking first in both harvested area (16,860 ha) and total production (39,650 tons) [4]. The dried fig sector is the principal driver of the fig industry, accounting for nearly 77% of global fig trade volume [5,6]. Extremadura represents the core of Spanish fig production, hosting more than 57% of the country’s cultivated area [6]. The region is particularly renowned for high-quality cultivars such as the ‘Calabacita’, which have strengthened Spain’s position as the leading European exporter to demanding markets such as France and Germany [6,7]. Within Extremadura, the Jerte Valley constitutes a distinctive production enclave where fig crops represent a key socio-economic activity, largely based on traditional cultivars finely adapted to a unique Mediterranean mountain microclimate [7,8].

Despite the species’ notable resilience, fig fruits are highly susceptible to fungal infection [8]. Their soft texture, high hygroscopicity, elevated sugar concentration, and ostioles (natural openings at the fruit base) make figs an ideal substrate for airborne pathogens, such as *Aspergillus* spp. and other mycotoxigenic fungi [1,2]. Within the *Aspergillus* genus, sections *Flavi* and *Nigri* are of major concern for dried fig contamination. Section *Flavi* includes the main producers of aflatoxins (AF), such as *A. flavus*, *A. parasiticus*, and related species [9]; whereas section *Nigri* comprises the primary producers of ochratoxin A (OTA), such as *A. niger*, *A. welwitschiae*, and others [10,11,12]. Both mycotoxin groups pose serious risks to human health and are therefore tightly regulated internationally. AF are of particular concern due to their potent carcinogenic, hepatotoxic, and genotoxic properties [13]. In general, there are more than 20 different AF, with AFB_1_, AFB_2_, AFG_1_, and AFG_2_ being the most important; specifically, AFB_1_ is classified as a Group 1 carcinogen by the International Agency for Research on Cancer (IARC) [14]. Furthermore, OTA is a serious hazard, classified by the IARC as a possible human carcinogen (Group 2B), and recognized for its nephrotoxic, immunotoxic, and teratogenic effects [14,15,16]. In response to these hazards, the EU has established maximum limits for AF (6 µg/kg for AFB_1_; 10 µg/kg for the sum of AFB_1_, AFB_2_, AFG_1_, and AFG_2_), and, for the first time, for OTA (8 µg/kg) in dried figs under Regulation (EU) 2023/915. The introduction of a specific OTA limit represents a substantial strengthening of the regulatory framework and poses an additional compliance challenge for producers, highlighting the need for improved pre-harvest risk management strategies.

**Figure 1:**
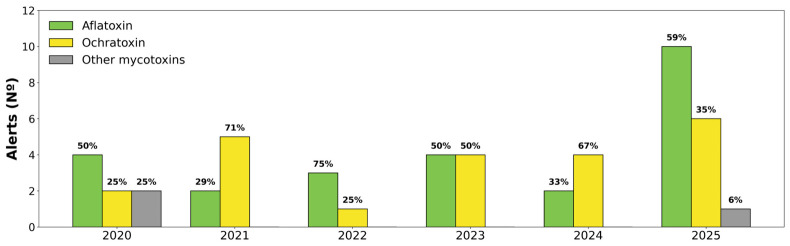
Temporal distribution of mycotoxin alerts in Spanish dried fruit and related products (2020–2025). Annual number of alerts reported through the Rapid Alert System for Food and Feed (RASFF), categorized by mycotoxin type: aflatoxins (AF, green), ochratoxins (OTA, yellow), and other mycotoxins (grey); percentages indicate the relative proportion of each type per year. Data source: RASFF database (products of Spanish origin). Prior to 2023, no EU maximum level for OTA applied to dried figs; OTA notifications recorded during that period were either information notifications or co-reported alongside AF exceedances. Commodity-specific breakdown by year is shown in Figure S1.

The impact of this regulation is illustrated by data from the Rapid Alert System for Food and Feed (RASFF). From 2020 to 2025, 25 alerts of AF contamination were reported in products originating from Spain, of which 32% involved figs (Figure 1). OTA alerts followed a similar pattern, with 22 notifications recorded over the same period, 36% involving figs, including notifications preceding the establishment of a specific EU maximum limit for OTA in dried figs under Regulation (EU) 2023/915. Prior to 2023, OTA notifications were generally issued either in conjunction with concurrent AF exceedances, for which maximum levels were already in force, or as information notifications flagging elevated contaminant levels in the absence of a binding regulatory threshold. The annual distribution of alerts and their commodity-specific breakdown are shown in Figure S1, which displays both the temporal trend in alert frequency and the relative contribution of figs and other commodities to AF and OTA notifications each year. The recurrence of both mycotoxin groups across years and the predominance of figs among notified products underscore the contamination risks inherent to this crop. Although Spain has a markedly lower notification rate than other EU member states, mycotoxin contamination remains the industry’s main regulatory challenge and requires field-based integrated management strategies.

The *Aspergillus* cycle and subsequent AF contamination in tree crops are well known. In general, *Aspergillus* spp. survive as spores and sclerotia in soil debris, producing vast quantities of airborne conidia as temperature rises, which are dispersed mainly by wind into the canopy, where they find infection sites [17,18,19]. In the USA and several African countries, the most advanced and effective control strategy consists of the mass application of atoxigenic *A. flavus* strains [20,21,22]. Across countries, treated crops have been shown to contain substantially lower AF levels, usually reduced by more than 80% than untreated crops from neighboring fields; these biocontrol practices based on native atoxigenic *Aspergillus* isolates have been recently reviewed by Ortega-Beltran et al. [23]. Yet, no equivalent commercial biocontrol product exists within the EU. In this context, a rigorous characterization of cultivar susceptibility across phenological stages becomes critical for developing integrated management strategies for fig crops in Europe [20,21,22].

The Spanish dried fig industry relies primarily on three cultivars that differ markedly in morphological and quality attributes: ‘Calabacita’, the dominant commercial cultivar in Extremadura, valued for its high quality, heat tolerance, thin skin, and strong adaptation to rainfed conditions; ‘Cuello Dama Blanco’, the largest (avg. 38 g) and sweetest (28 °Brix), particularly suited for fresh consumption and premium dried fig production; and ‘Granito’, which is smaller (avg. 20 g, 24 °Brix) and of particular interest for dried fig processing given its local cultivation [24,25,26]. Despite these differences, all three cultivars exhibit a bifera growth habit, yellowish-green skin, and amber-colored pulp. Morphological traits (notably ostiole aperture) are likely to play a key role in determining fruit colonization by airborne *Aspergillus* spores [26,27,28]. Susceptibility to AF accumulation is further shaped by phenological stage. The “firm ripe” stage constitutes the period of highest susceptibility, coinciding with maximum fruit expansion, elevated water activity, and a sharp rise in sugar content [26,29]. Genetic factors may additionally govern cultivar-level differences in AF accumulation independently of surface colonization [30,31]. Elevated temperature and drought stress resulting from climate change can enhance the toxigenic potential of *Aspergillus* section *Flavi* [32,33,34], with predictive models indicating a potential expansion of toxigenic fungi under projected warming scenarios [33,35].

Despite the recognized importance of *Aspergillus* spp. in fig contamination, the relative contributions of cultivar-specific traits, infection pathways, and field-level factors to mycotoxin risk remain poorly quantified in Mediterranean production systems. Bridging this gap requires both mechanistic experiments that quantify susceptibility under controlled and field conditions, and industry-scale surveillance data that capture contamination outcomes under real production constraints.

We tested the following hypotheses: (i) cultivars differ in their susceptibility to *Aspergillus* colonization, with ostiole morphology and fruit size as putative structural determinants; (ii) dry conidial inoculation more closely approximates natural airborne infection conditions than aqueous suspension inoculation; (iii) cultivar susceptibility patterns observed under controlled conditions are reproducible under orchard conditions; and (iv) experimental susceptibility patterns observed under controlled and orchard conditions are consistent with mycotoxin contamination levels recorded in commercial batches from a multi-year industry surveillance dataset. The study combines a mechanistic plant pathology component, comprising controlled inoculation and orchard experiments, and a food safety surveillance component, involving multi-year industry monitoring of mycotoxin contamination in commercial batches. By integrating these lines, the study addresses a critical gap between the experimental understanding of infection processes and real-world mycotoxin risk in dried fig production. Elucidating these contamination dynamics provides the framework needed to reduce mycotoxin levels, prevent economic losses for farmers, and protect consumer health.

## 2. Materials and Methods

### 2.1. Cultivar Susceptibility to A. flavus Under Controlled Conditions (Experiment 1)

Fruits of *Ficus carica* L. from three cultivars representative of the Jerte Valley (‘Granito’, ‘Cuello Dama Blanco’, and ‘Calabacita’) were harvested from the experimental field of CICYTEX (Centro de Investigaciones Científicas y Tecnológicas de Extremadura) in Guadajira (Extremadura, Spain) during the 2022 and 2023 seasons. Fruit size classes were defined based on the equatorial diameter, which was measured at the widest transverse section of each fruit, perpendicular to its vertical axis. Size classes were categorized as follows: for ‘Granito’, small (16.65–20.69 mm), medium (23.61–25.97 mm), and large (33.81–34.35 mm); for ‘Cuello Dama Blanco’, small (16.29–19.47 mm), medium (27.48–31.67 mm), and large (37.03–39.12 mm); and for ‘Calabacita’, small (20.37–24.70 mm), medium (28.38–32.53 mm), and large (37.91–40.77 mm).

In 2022, ‘Cuello Dama Blanco’ and ‘Calabacita’ were evaluated. For each cultivar, 50 fruits per size class were collected on the same date and inoculated with unsealed ostioles. In 2023, the trial was expanded to include all three cultivars, with the addition of ‘Granito’, and to compare two ostiole treatments: (i) ostioles sealed with molten silicone and (ii) ostioles left unsealed. For each cultivar × size × ostiole treatment combination, experiments were conducted in triplicate, with each group including a non-inoculated control.

In both seasons, two *A. flavus* strains (ASP40 and ASP41) from the *Aspergillus* collection of the University of Cordoba (EPICON Lab) were used; both had been isolated from dried figs between 2019 and 2024, as described by Janfi et al. (2025) [9]. Seven to ten fruits per size class within each replicate were placed in plastic containers that served as humid chambers and were sprayed with a conidial suspension (10^5^ conidia mL^−1^) prepared from these strains. All inoculation and handling procedures were carried out in a biosafety cabinet under sterile conditions to prevent cross-contamination. Following inoculation, fruits were incubated in the dark at 30 °C for 5 days.

After incubation, fruits were surface disinfected by immersion in 0.5% sodium hypochlorite for 1 min, rinsed with sterile distilled water, and aseptically sectioned. Five internal tissue fragments (4 × 4 × 4 mm) per fruit, comprising internal inflorescence and underlying receptacle tissue, were cut out using a sterile scalpel and plated onto salt agar (60 g NaCl L^−1^). This medium was used to promote the growth and sporulation of *Aspergillus* spp. while inhibiting competing microorganisms. Plates were incubated at 30 °C for an additional 5 days. Colonies were identified as *A. flavus* based on morphological characteristics (e.g., yellow-green radiate conidial heads, globose to sub-globose vesicles, and finely echinulate conidia), following standard taxonomic criteria [36]. Internal fruit colonization was quantified as the proportion of colonized internal tissue fragments (0–5 per fruit). In addition, colonization incidence (0 or 1) was calculated as the proportion of fruits internally colonized by the pathogen, regardless of the number of positive fragments. This approach enabled a quantitative comparison of fungal colonization among cultivars, fruit sizes, and ostiole treatments.

### 2.2. Efficacy of Dry vs. Aqueous Conidial Suspension Inoculation Methods (Experiment 2)

Inoculation assays using aqueous conidial suspensions are widely used under controlled conditions to assess *Aspergillus* infection under optimal moisture availability. However, their transfer to orchard conditions is operationally challenging because conidial suspensions may germinate prematurely at high temperatures and during field handling, potentially compromising inoculum viability. Therefore, dry inoculation was evaluated as an alternative approach to better approximate field conditions. In 2024, mature fruits of ‘Calabacita’ were used to compare dry and aqueous *A. flavus* inoculation methods. Each treatment comprised three independent replicates conducted on separate occasions using freshly prepared inoculum, each consisting of five inoculated humid chambers and one non-inoculated control chamber, with 10 fruits per chamber (150 inoculated and 30 non-inoculated fruits per treatment). Fruits were sprayed with an aqueous conidial suspension of *A. flavus* (10^5^ conidia mL^−1^; Tween-20 at 0.01% *v*/*v* as a surfactant), avoiding droplet runoff while ensuring full surface coverage. For the dry inoculation method, the same number of fruits and chambers were used. Conidial powder of isolates ASP40 and ASP41, obtained from 7-day-old sporulating cultures grown on Potato Dextrose Agar (PDA), was dispersed over surface-disinfected fruits using a PVC tube connected to a compressed-air tire inflator. Deposition density was estimated by placing adhesive glass slides within the inoculation area and examining them at 400× magnification, yielding a mean deposition density of 148 ± 38 spores mm^−2^, with one slide per chamber. Although originally developed for powdery mildew inoculation in cereals [18], this approach has not been previously reported for *Aspergillus* inoculation in fruit crops. Following inoculation, all fruits were incubated at 30 °C for 5 days. Colonization was assessed using the same surface disinfection, tissue plating, and incubation procedures described in Section 2.1.

### 2.3. Field Assays

#### 2.3.1. Colonization of Canopy vs. Ground Fruits by *A. flavus* (Experiment 3)

The field trial was conducted at the CICYTEX experimental field in Guadajira (Extremadura, Spain) during the 2025 season (July–August). Three rows per cultivar (‘Calabacita’, ‘Cuello Dama Blanco’, and ‘Granito’) were selected, and within each row, three 5-year-old trees were inoculated with dry conidia of isolates ASP40 and ASP41.

Inoculation was performed for 1 min per tree using a PVC tube connected to a compressed-air tire inflator (40–50 L min^−1^) containing a Petri dish with a 7-day-old sporulating culture grown on PDA, to facilitate conidial dispersal throughout the tree canopy. The same inoculation protocol as described for Experiment 2 was used; furthermore, conidial deposition density was verified to be comparable (100–150 conidia mm^−2^).

Before inoculation, the soil beneath the canopy of each tree was carefully cleaned to remove previously fallen fruits and ensure that fruits subsequently collected from the ground corresponded to fruits exposed to the inoculation treatment during the experiment. In addition, three non-inoculated trees per cultivar, distributed across different rows, were included as controls.

Fruit sampling was conducted 25 days after inoculation, with fruits collected separately from the canopy and from the ground beneath each tree. At sampling, 25 fruits per tree and cultivar were collected and processed according to the laboratory protocol described in Section 2.1, yielding 75 fruits per cultivar × position combination (3 rows × 3 trees × 25 fruits per tree).

Colonies belonging to *Aspergillus* section *Flavi* (reflecting both the applied *A. flavus* inoculum and naturally occurring populations) and *Aspergillus* section *Nigri* (naturally present in the orchard and not subjected to inoculation) were quantified based on colony morphology.

#### 2.3.2. *A. flavus* Detection on Leaf Surfaces

To assess the presence of *A. flavus* on vegetative tissues, leaf samples were collected from each of the three inoculated trees per cultivar, transported to the laboratory, and pooled into batches of five leaves per tree. Each batch was weighed and washed in sterile water containing 0.001% Tween-20 (*v*/*v*) to facilitate spore detachment. The wash solution was centrifuged at 10,000 rpm for 10 min, and the resulting pellet was resuspended in 2 mL of sterile water. Aliquots of 100 µL were plated in triplicate onto *Aspergillus flavus-parasiticus agar* (AFPA) selective medium per tree and incubated at 30 °C for 5 days. Colonies were identified based on typical morphological characteristics and their colorimetric reaction on AFPA, and fungal load was expressed as colony-forming units per gram of fresh leaf tissue (CFU g^−1^), considering the dilution factor and initial sample weight. Plate counts were averaged within each tree to obtain a single tree-level value.

### 2.4. Mycotoxin Contamination Data from Industry Surveillance

**Data curation.** Mycotoxin records from a local fig-processing cooperative surveillance covering the 2014–2023 marketing seasons were obtained from annual laboratory reports generated by Eurofins (www.eurofins.com), an accredited laboratory routinely applying standardized LC-MS/MS-based methods for regulatory mycotoxin determination. These records were consolidated into a single dataset comprising 1560 replicate records corresponding to 568 commercial batches. Records were screened for structural integrity: rows lacking a valid harvest year or replicate code, as well as samples corresponding to non-fig commodities, were excluded. Numerical concentrations of AFB_1_, AFB_2_, AFG_1_, AFG_2_, total aflatoxins (AFTA), and OTA were parsed according to standard censoring conventions. Left-censored values reported as “<LOQ” or as qualitative non-detection codes were imputed as LOQ/2 (medium-bound substitution), whereas right-censored values (“>upper-LOQ”) were retained at the reported boundary value. Batches were classified as non-compliant when the maximum replicate-level concentration exceeded the applicable maximum level as reported by the accredited laboratory, without correction for measurement uncertainty.

When AFTA was not reported, but its individual AF were, AFTA was reconstructed as the sum of AFB_1_ + AFB_2_ + AFG_1_ + AFG_2_. Replicate-level results were aggregated to the batch level by taking the maximum across replicates. After cleaning and aggregation, the consolidated dataset comprised 568 unique batches. For the present analysis, batches were restricted to ‘Granito’ and ‘Calabacita’, the two cultivars with multi-year representation in the surveillance records. Batches from minor cultivars or with single-year representation were excluded (*n* = 242), yielding a final analytical dataset of 326 batches (‘Granito’, *n* = 256, harvest years 2014–2023; ‘Calabacita’, *n* = 70, harvest years 2020–2023). To ensure temporal comparability between cultivars, the main analyses and figures were restricted to the period 2020–2023, for which both cultivars were represented (‘Granito’, *n* = 114; ‘Calabacita’, *n* = 70). The complete year-by-year record for ‘Granito’ across the full surveillance period (2014–2023, *n* = 256) is provided in Supplementary Table S1.

**Regulatory evaluation.** Each batch was evaluated against the maximum levels (ML) established in Commission Regulation (EU) 2023/915 for dried figs: AFB_1_ > 6 µg/kg, AFTA > 10 µg/kg, OTA > 8 µg/kg. For batches from harvest years prior to 2023, the OTA maximum level was not yet in force; its application here is retrospective and intended solely as a risk assessment exercise, not as an indication of historical legal non-compliance. A batch was classified as non-compliant whenever any of the three concentrations exceeded its corresponding ML. Among non-compliant batches, the putative producing taxon was provisionally inferred from the mycotoxin profile, as individual isolates were not available for molecular identification or isolate-level toxin confirmation. Specifically, batches exceeding the OTA limit were tentatively attributed to *Aspergillus* section *Nigri* (as OTA production is typically associated with species within this section); among the remaining non-compliant batches, those with detectable AFG_1_ and/or AFG_2_ were presumptively attributed to *A. parasiticus*, and those with only AFB profiles to *A. flavus*. A small number of batches exceeding AFTA without individually quantified AFB or AFG were assigned to *A. flavus* by default in the absence of detectable AFG. This profile-based attribution should be considered indicative rather than definitive. These analyses are part of an industry quality assurance system whereby non-compliant batches are identified and removed before reaching consumers. The dataset presented here, therefore, reflects the contamination status before such elimination.

**Visualization of non-compliance co-occurrence.** Co-occurrence of AFB_1_, AFTA, and OTA exceedances across batches was visualized using Venn diagrams, stratified by cultivar. A summary table detailing non-compliance counts, proportions by year and cultivar, and species attribution was generated and provided as Supplementary Material.

**Mycotoxin concentration distributions among detectable batches.** For each toxin, the concentration distribution was characterized within the subset of batches with quantifiable values (i.e., excluding left-censored samples). Medians and ranges were summarized by cultivar, and between-cultivar comparisons of the full distributions were performed (see Section 2.5 for statistical details).

### 2.5. Statistical Analysis

All statistical analyses were conducted in Python (v. 3.12.3) using statsmodels (v. 0.14.6), scipy (v. 1.17.1), and matplotlib-venn (v. 1.1.2) libraries.

**Experiment 1 (Controlled conditions).** Differences in the severity and incidence of *Aspergillus* section *Flavi* colonization among cultivars and ostiole treatments were analyzed separately for each experimental year to focus the statistical analysis on independent variables rather than seasonal variation. Severity (percentage of colonized internal fruit fragments per fruit, based on five fragments assessed per fruit) was analyzed using ordinary least squares (OLS) regression on arcsine square-root transformed values (arcsin(√p)). Incidence (colonized vs. non-colonized fruit) was analyzed using generalized linear models (GLMs) with a binomial distribution and a logit link function.

In the 2023 experiment, fruit size (small = 1, medium = 2, large = 3) was included as a continuous covariate because preliminary analyses indicated that susceptibility increased with fruit size. The effect of fruit size was evaluated using the t-statistic (OLS, severity) or z-statistic (binomial GLM, incidence) associated with the corresponding coefficient (β), which represents the expected change in the response variable per one-unit increment in fruit size category. For incidence, odds ratios were calculated as exp (β).

In 2023, ostiole treatment (sealed vs. unsealed) and its interaction with cultivar were included as fixed factors, whereas in 2022 only cultivar was included, as no ostiole treatment was applied. In both years, replicate (*n* = 3) was included as a fixed blocking factor in all models. Pairwise comparisons among cultivar × ostiole-treatment combinations were performed using Fisher’s protected LSD test for severity and Wald z-tests for incidence at α = 0.05. Compact letter displays (CLD) were used to indicate statistically homogeneous groups, with uppercase letters representing severity and lowercase letters representing incidence.

**Experiment 2 (Dry vs. aqueous conidial suspension inoculations).** Severity, expressed as the percentage of colonized internal fragments per fruit, was compared between dry conidial inoculation and inoculation using an aqueous conidial suspension by means of the Mann–Whitney U test, because severity values did not meet normality assumptions and showed an excess of zeros, particularly under the aqueous suspension treatment. Incidence, defined as the proportion of colonized fruits, was compared between inoculation methods using Fisher’s exact test, given the binary nature of the response variable and the 2 × 2 contingency table structure. In both analyses, the individual fruit was the unit of observation; data from all inoculated chambers within each treatment were pooled (*n* = 150 fruits per treatment). Chambers within each replicate received inoculum prepared in the same session and functioned as logistical containers rather than independent experimental units. Treatment was assigned at the replicate-run level, and variation among chambers was therefore nested within replicate runs.

**Experiment 3 (Field assays).** Analyses were conducted at the individual fruit level (*n* = 75 fruits per cultivar × position combination). To compare canopy and ground fruits, differences in the severity and incidence of colonization by *Aspergillus* sections *Flavi* and *Nigri* among cultivars and fruit positions (canopy vs. ground) were analyzed using generalized linear models (GLMs) with a binomial distribution and a logit link function. Cultivar, fruit position, and their interaction were included as fixed factors in all models.

Severity was analyzed as the number of colonized internal fruit fragments out of five fragments assessed per fruit, whereas incidence was analyzed as colonized vs. non-colonized fruit. Pairwise comparisons among cultivar × fruit-position combinations were performed using Wald z-tests at *p ≤* 0.05. Compact letter displays (CLD) were used to summarize statistically homogeneous groups.

For detection of *A. flavus* on leaf surfaces, plate counts were averaged within each tree to obtain a single tree-level value. These tree-level means (*n* = 3 trees per cultivar) were compared among cultivars using the Kruskal–Wallis test, followed by Dunn’s multiple-comparison test at *p ≤* 0.05.

**Industry surveillance data.** Mycotoxin exceedance rates were calculated relative to the maximum levels established in Commission Regulation (EU) 2023/915. Replicate-level observations were aggregated to the batch level by retaining the maximum value across replicates, following a conservative criterion for regulatory assessment. Co-occurrence of AFB_1_, AFTA, and OTA exceedances among batches was visualized using Venn diagrams generated with the matplotlib-venn library.

A summary table detailing non-compliance counts, yearly proportions by cultivar, inferred fungal attribution, and results of pairwise statistical comparisons was generated and provided as Supplementary Material. Pairwise comparisons of proportions among years within each cultivar and toxin/species category were performed using Zar’s multiple-comparison test for proportions [37], based on the Tukey-type q statistic from the studentized range distribution (k groups, df = ∞). To avoid inconsistencies in compact letter displays (CLD) caused by unequal sample sizes, years sharing identical proportions collapsed into the same comparison block prior to testing, ensuring that identical proportions always shared the same letter. Lowercase letters denote the ranking of proportions from highest to lowest, whereas shared letters indicate non-significant differences at *p* ≤ 0.05. Between-cultivar differences in non-compliance proportions (pooled across all years within cultivar; ‘Granito’ 2014–2023, *n* = 256; ‘Calabacita’ 2020–2023, *n* = 70) were evaluated for each toxin/species category using Pearson’s χ^2^ test with Yates’ continuity correction for 2 × 2 contingency tables. Fisher’s exact test was used whenever expected cell frequencies were below 5 (Cochran’s rule). Uppercase letters denote significant differences between cultivars at *p* ≤ 0.05.

For mycotoxin concentration distributions among detectable batches (excluding left-censored observations), departure from log-normality within each cultivar was evaluated using the Shapiro–Wilk test on log_10_-transformed concentrations. Differences between cultivars were assessed using the two-sample Anderson-Darling test [38] applied to raw concentration values, as this test is sensitive to differences across the entire distribution, including the upper tails relevant to regulatory exceedance. Kernel density plots of log_10_-transformed concentrations, back-transformed to the linear scale, were generated to visualize cultivar-specific distribution patterns. Because batches exceeding regulatory thresholds are systematically removed during industrial processing, the dataset provides a conservative estimate of field-level mycotoxin contamination prior to commercial exclusion procedures.

## 3. Results

### 3.1. Cultivar Susceptibility to A. flavus Under Controlled Conditions (Experiment 1)

**Figure 2:**
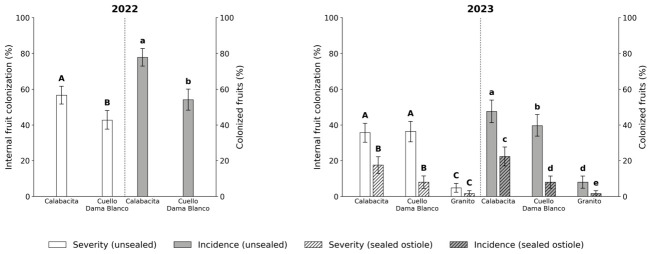
Colonization of fig fruits (*Ficus carica* L.) by *Aspergillus flavus* in 2022 (**left** panel) and 2023 (**right** panel). Within each year, severity (mean percentage of colonized internal fruit fragments, left *Y*-axis, white bars) is shown to the left of the dotted divider, and incidence (percentage of colonized fruits, right *Y*-axis, grey bars) to the right. Error bars indicate the standard error of the mean. In 2023, hatched and unhatched bars correspond to sealed and unsealed ostioles, respectively. Uppercase and lowercase letters indicate significant differences in severity and incidence, respectively (α = 0.05). Severity: OLS regression on arcsine square-root transformed values; incidence: binomial GLM. Fixed factors: cultivar and, in 2023, ostiole treatment and their interaction; fruit size as a covariate; replicate as a blocking factor.

In 2022, both the severity and incidence of internal colonization by *A. flavus* were significantly higher in ‘Calabacita’ than in ‘Cuello Dama Blanco’ (severity: F_1,139_ = 4.62, *p* = 0.033; incidence: χ^2^ = 9.43, df = 1, *p* = 0.002; Figure 2, left panel). Mean severity reached 56.7% in ‘Calabacita’ and 42.8% in ‘Cuello Dama Blanco’, whereas incidence was 77.8% and 54.2%, respectively.

In 2023, fruit size was included as a continuous covariate in all models because preliminary analyses indicated increased susceptibility with increasing fruit size. Fruit size emerged as a significant positive predictor of both severity (β = 11.83, SE = 2.01, t = 5.90, *p* < 0.001) and incidence (β = 1.14, SE = 0.20, z = 5.59, *p* < 0.001). For incidence, the estimated odds ratio was 3.13 per increment in fruit size category, indicating that the odds of colonization increased more than threefold with each increase in fruit size category. Mean severity increased monotonically from 4.9% in small fruits to 18.3% and 28.6% in medium and large fruits, respectively, whereas incidence increased from 6.3% to 23.0% and 34.1%, respectively.

After adjustment for fruit size, significant differences among cultivars and ostiole treatments were detected. A significant cultivar × ostiole-treatment interaction was observed for severity (F_2,369_ = 4.75, *p* = 0.009; Figure 2, right panel). Pairwise comparisons resolved three statistically distinct groups (indicated by CLD letters in Figure 2, right panel): ‘Calabacita’ and ‘Cuello Dama Blanco’ showed the highest severity values (35.6% and 36.2%, respectively), whereas sealing the ostiole markedly reduced colonization in both cultivars (17.5% and 7.9%, respectively), forming a second group. In contrast, ‘Granito’ (the least susceptible cultivar) consistently showed the lowest severity values regardless of ostiole treatment (unsealed: 4.8%; sealed: 1.6%), forming the third group.

For incidence, the cultivar × ostiole-treatment interaction was not significant (χ^2^ = 1.79, df = 2, *p* = 0.41), although both cultivar (χ^2^ = 50.73, df = 2, *p* < 0.001) and ostiole treatment (χ^2^ = 35.32, df = 1, *p* < 0.001) had highly significant effects. Pairwise comparisons resolved five statistically distinct groups (indicated by CLD letters in Figure 2, right panel): ‘Calabacita’-unsealed (47.6%), ‘Cuello Dama Blanco’-unsealed (39.7%), ‘Calabacita’-sealed (22.2%), ‘Cuello Dama Blanco’-sealed and ‘Granito’-unsealed (both 7.9%), and ‘Granito’-sealed (1.6%).

### 3.2. Efficacy of Dry vs. Aqueous Conidial Suspension Inoculation Methods (Experiment 2)

**Figure 3:**
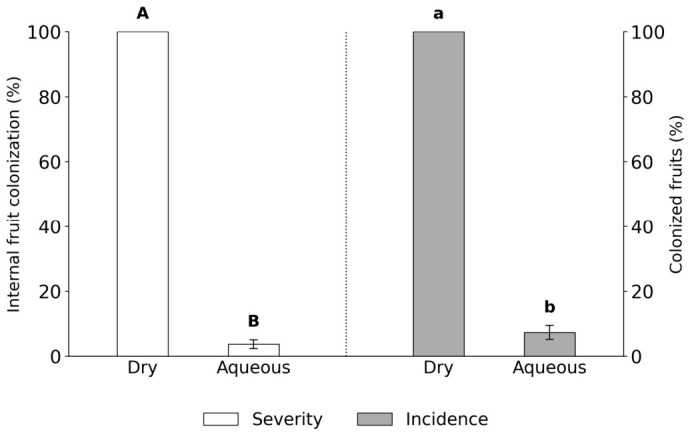
Colonization of ‘Calabacita’ fig fruits (*Ficus carica* L.) by *Aspergillus flavus* following dry or aqueous conidial inoculation. Severity (left, white bars): mean percentage of colonized internal fruit fragments; incidence (right, grey bars): percentage of colonized fruits. Error bars: standard error of the mean; *n* = 150 fruits per treatment. Uppercase and lowercase letters indicate significant differences in severity (Mann–Whitney U test) and incidence (Fisher’s exact test), respectively (*p* < 0.05).

Dry inoculation with *A. flavus* conidia resulted in substantially higher colonization of ‘Calabacita’ fig fruits than inoculation using an aqueous conidial suspension for both severity and incidence (Figure 3). Mean severity reached 100% (±0 SEM) under dry conidial inoculation, compared with 3.7% (±1.3 SEM) under inoculation with the aqueous conidial suspension (Mann–Whitney U test, *p* < 0.001; *n* = 150 inoculated fruits per treatment). Similarly, incidence was markedly higher under dry inoculation, with 100% of fruits colonized compared with 7.3% under inoculation using the aqueous conidial suspension (Fisher’s exact test, *p* < 0.001).

### 3.3. Varietal Susceptibility Under Orchard Conditions (Experiment 3)

**Figure 4:**
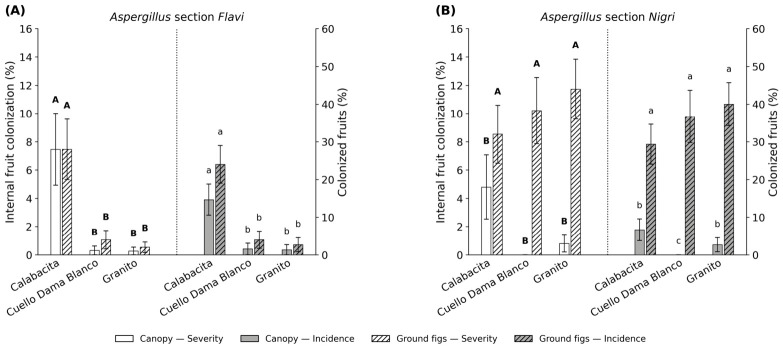
Colonization of fig fruits (*Ficus carica* L.) by *Aspergillus* section *Flavi* (**A**) and section *Nigri* (**B**) under field conditions following dry conidial inoculation. Within each panel, severity (mean percentage of colonized internal fruit fragments, left *Y*-axis, white bars) is shown to the left of the dotted divider, and incidence (percentage of colonized fruits, right *Y*-axis, grey bars) to the right. Hatched and unhatched bars correspond to ground and canopy fruits, respectively. Error bars: standard error of the mean. Uppercase and lowercase letters indicate significant differences in severity and incidence, respectively (*p* < 0.05).

Field inoculation with *A. flavus* (section *Flavi*) revealed contrasting susceptibility patterns for colonization by sections *Flavi* and *Nigri* (Figure 4). Sample sizes per cultivar × fruit-position combination ranged from 65 to 75 fruits for section *Flavi* (*n* = 440 total) and from 49 to 75 fruits for section *Nigri* (*n* = 414 total). For section *Flavi*, cultivar significantly affected both severity and incidence (χ^2^ = 16.99, df = 2, *p* < 0.001; χ^2^ = 9.95, df = 2, *p* = 0.007, respectively). ‘Calabacita’ consistently showed higher values than ‘Cuello Dama Blanco’ and ‘Granito’, which did not differ significantly from each other (Figure 4A). In contrast, neither fruit position (canopy vs. ground-collected fruits) nor the cultivar × position interaction significantly affected section *Flavi* colonization (position—severity: χ^2^ = 1.09, df = 1, *p* = 0.296; incidence: χ^2^ = 2.06, df = 1, *p* = 0.151; interaction for severity: χ^2^ = 0.34, df = 2, *p* = 0.844; incidence: χ^2^ = 0.09, df = 2, *p* = 0.955).

For section *Nigri*, the pattern was inverted. The cultivar did not significantly affect either severity or incidence (χ^2^ = 2.72, df = 2, *p* = 0.257; χ^2^ = 1.26, df = 2, *p* = 0.532, respectively), whereas fruit position significantly affected both response variables (severity: χ^2^ = 8.18, df = 1, *p* = 0.004; incidence: χ^2^ = 11.12, df = 1, *p* < 0.001). Fruits collected from the ground consistently exhibited higher colonization by section *Nigri* species than canopy fruits across all three cultivars (Figure 4B), with the largest differences observed for incidence. The cultivar × position interaction was not significant for either severity or incidence (χ^2^ = 5.14, df = 2, *p* = 0.076 and χ^2^ = 2.42, df = 2, *p* = 0.298, respectively).

The inoculum load on the leaf surface of inoculated trees, monitored to verify successful inoculation, was significantly higher in ‘Calabacita’ (24.2 ± 10.3 CFU g^−1^ leaf) than in ‘Granito’ (4.8 ± 2.5 CFU g^−1^ leaf) and ‘Cuello Dama Blanco’ (1.4 ± 0.9 CFU g^−1^ leaf), although differences among cultivars were not significant according to the Dunn test at *p* ≤ 0.05.

### 3.4. Mycotoxin Contamination in Commercially Processed Dried Figs: Industry Surveillance Data (2020–2023)

**Figure 5:**
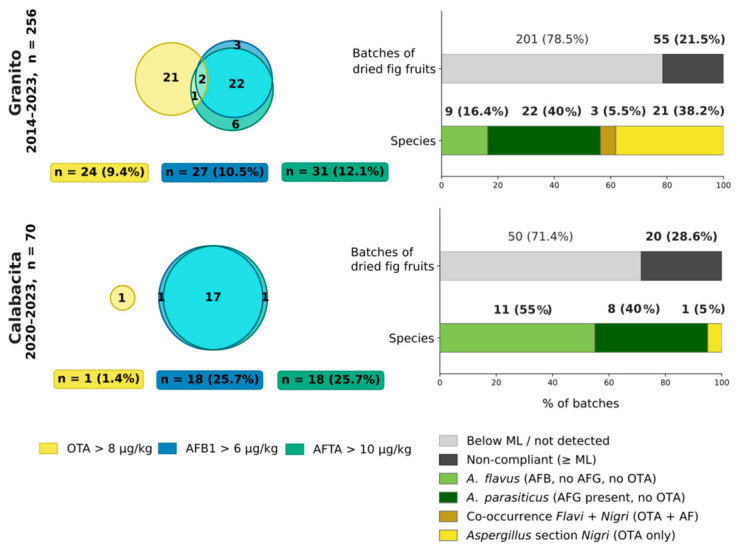
Co-occurrence of mycotoxin limit exceedances in commercial dried fig batches of ‘Granito’ (2020–2023; *n* = 114) and ‘Calabacita’ (2020–2023; *n* = 70) monitored through the industry surveillance program. Left panels: Venn diagrams showing batches exceeding the maximum levels established by Regulation (EU) 2023/915 for ochratoxin A (OTA ≥ 8 µg kg^−1^), aflatoxin B_1_ (AFB_1_ ≥ 6 µg kg^−1^), and total aflatoxins (AFTA ≥ 10 µg kg^−1^). The OTA threshold was applied retrospectively across the full surveillance series. Numbers within diagram sectors indicate exclusive or overlapping exceedances. Right panels: proportion of non-compliant batches and inferred fungal attribution. Upper bars: proportion of batches exceeding at least one regulatory limit (dark grey) relative to total analyzed (light grey). Lower bars: batches tentatively attributed to *Aspergillus flavus* (AFB only, no detectable AFG), *A. parasiticus* (AFG detected), co-occurrence of section *Flavi* and *Nigri*, or section *Nigri* (OTA only). Attribution was inferred from mycotoxin profiles and should be considered indicative rather than definitive. Non-compliant batches were removed prior to commercialization.

Industry surveillance data from a local fig-processing cooperative, generated through routine pre-commercialization screening as part of its quality assurance program, provided a real-world counterpart to the susceptibility patterns observed under controlled conditions. A total of 184 commercial batches from the cultivars ‘Granito’ (*n* = 114) and ‘Calabacita’ (*n* = 70) were analyzed (harvest years 2020–2023). Overall, 37 ‘Granito’ batches (32.5%) and 20 ‘Calabacita’ batches (28.6%) exceeded at least one regulatory maximum level (ML) established by Commission Regulation (EU) 2023/915 for AFB_1_ (>6 µg/kg), AFTA (>10 µg/kg), or OTA (>8 µg/kg) (Figure 5). For batches harvested before 2023, application of the OTA maximum level is retrospective and should not be interpreted as historical legal non-compliance.

In ‘Granito’, AFTA exceedances were the most frequent (21.9%, *n* = 25), followed by AFB_1_ (18.4%, *n* = 21) and OTA (10.5%, *n* = 12). Species attribution based on the mycotoxin profile indicated that *A. parasiticus* (AFG detected) accounted for 17 batches (45.9% of all non-compliant batches), *Aspergillus* section *Nigri* (OTA only, without AF exceedance) for 9 batches (24.3%), and *A. flavus* (AFB only, without detectable AFG) for 8 batches (21.6%). In addition, the co-occurrence of aflatoxigenic section *Flavi* species and OTA-producing section *Nigri* species was inferred for 3 batches (8.1%).

In contrast, ‘Calabacita’ exhibited markedly higher AF-related non-compliance. AFB_1_ and AFTA exceedances each reached 25.7% (*n* = 18 for both), whereas OTA exceedance was rare (1.4%, *n* = 1). The Venn diagram (Figure 5, left panel) shows that AFB_1_ and AFTA exceedances largely overlapped (17 batches), with only one batch exceeding AFB_1_ alone and one exceeding AFTA alone. Species attribution among non-compliant batches indicated that *A. flavus* accounted for 55.0% of cases, *A. parasiticus* for 40.0%, and *Aspergillus* section *Nigri* for 5.0%. It should be noted that all species attributions in this section are inferred from mycotoxin profiles and should be interpreted as indicative of the likely fungal contributors rather than as confirmed identifications.

**Figure 6:**
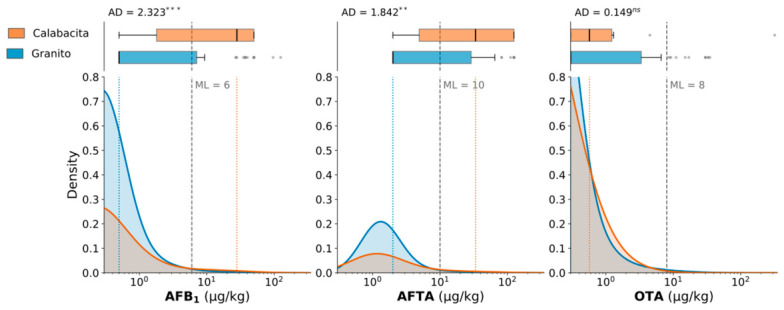
Distribution of aflatoxin B_1_ (AFB_1_), total aflatoxins (AFTA), and ochratoxin A (OTA) concentrations (µg/kg) in commercial batches of ‘Calabacita’ and ‘Granito’ (harvest years 2020–2023). Each panel combines a horizontal boxplot (top) and a kernel density estimate (bottom), plotted on a log_10_-transformed x-axis. Sample sizes (*n*) for AFB_1_, AFTA, and OTA, respectively, are ‘Calabacita’ (*n* = 29, 27, 12) and ‘Granito’ (*n* = 75, 73, 66). Dotted vertical lines indicate the median (M) for each cultivar; dashed vertical lines indicate the regulatory maximum level (ML) established by Regulation (EU) 2023/915 for dried figs. Distributions between cultivars were compared using the two-sample Anderson-Darling (AD) test; significance is indicated as *p* < 0.05 *, *p* < 0.01 **, *p* < 0.001 ***; ns, not significant.

Figure 6 presents the distribution of mycotoxin concentrations in batches with quantifiable values (i.e., excluding left-censored samples) for the comparable period 2020–2023, combining kernel density estimates and horizontal boxplots. For AFB_1_, ‘Calabacita’ showed a markedly higher median concentration (28.0 µg kg^−1^) than ‘Granito’ (0.5 µg kg^−1^), and the two-sample Anderson-Darling test confirmed significant differences between cultivar distributions (AD = 2.323, *p* < 0.001). Similar trends were observed for AFTA, with median concentrations of 33.6 µg kg^−1^ in ‘Calabacita’ and 2.0 µg kg^−1^ in ‘Granito’ (AD = 1.842, *p* = 0.002).

In contrast, quantifiable OTA values were more frequent in ‘Granito’ (*n* = 66, median = 0.3 µg kg^−1^) than in ‘Calabacita’ (*n* = 12, median = 0.57 µg kg^−1^), although OTA distributions did not differ significantly between cultivars (AD = 0.149, *p* = 0.779), consistent with the low OTA exceedance rate observed in ‘Calabacita’.

Supplementary Table S1 provides a year-by-year breakdown of non-compliant batches by cultivar, toxin, and putative producing species, together with statistical comparisons (lowercase letters indicate among-year differences within cultivars, whereas uppercase letters indicate between-cultivar comparisons across the full surveillance period). Between-cultivar comparisons revealed that ‘Calabacita’ had significantly higher proportions of AFB_1_ exceedances than ‘Granito’ (15.7% vs. 3.5%; B vs. A), as well as higher proportions of AFTA exceedances (14.3% vs. 3.1%; B vs. A). Conversely, ‘Granito’ showed a significantly higher proportion of OTA exceedances (9.4% vs. 1.4%; A vs. B). No significant differences between cultivars were detected for *A. parasiticus* attribution (both AFB_1_ and AFTA columns) or for total non-compliance (≥1 toxin limit), although total non-compliance was numerically higher in ‘Calabacita’ (28.6% vs. 21.5%).

Overall, the industrial surveillance data were consistent with the susceptibility patterns observed in the controlled inoculation experiments, with ‘Granito’ showing lower AF-related contamination than ‘Calabacita’.

## 4. Discussion

Across all experimental systems, ‘Calabacita’ consistently exhibited the highest susceptibility to colonization by *Aspergillus* section *Flavi*, representing the most robust and agronomically relevant finding of this study. This susceptibility ranking remained remarkably consistent under controlled inoculations, field assays, and long-term industrial surveillance, suggesting that cultivar-dependent differences may be substantially determined by intrinsic fruit traits, although the contribution of environmental and management factors cannot be excluded based on the present data. These observations agree with previous studies suggesting that fig susceptibility to fungal colonization is strongly influenced by structural integrity and biochemical characteristics of the fruit [7,39]. Fruit size further modulated colonization risk, with larger fruits showing greater susceptibility than medium and small calibers. This pattern is consistent with previous observations linking larger ostiolar apertures to enhanced fungal invasion in figs [40]. Importantly, the period of maximum fruit susceptibility coincides with the seasonal peaks of airborne *Aspergillus* spores detected in our aerobiological studies, which typically occur between mid-August and early September under Mediterranean conditions (Janfi et al., unpubl. data). The temporal synchronization between peak inoculum availability and increased fruit susceptibility may therefore play a critical role in determining the final contamination risk in dried figs. Because the ostiole represents the only natural external opening of the syconium, it likely constitutes the principal infection gateway for aflatoxigenic *Aspergillus* species.

The ostiole-sealing experiment provided mechanistic support for this hypothesis. Physically blocking the ostiole substantially reduced colonization in all cultivars and, importantly, eliminated the cultivar-dependent differences observed under unsealed conditions. This finding strongly suggests that differential susceptibility is primarily associated with ostiolar accessibility and morphology rather than with post-penetration resistance mechanisms, which is consistent with Michailides (1998) [40], who identified ostiole morphology and integrity as major determinants of fungal invasion in figs. Furthermore, physiological disorders such as ostiole-end splitting and lateral cracking can substantially increase fungal ingress during fruit ripening [41]. The high susceptibility observed in ‘Calabacita’ may therefore partly reflect an increased ostiolar opening or a greater tendency toward splitting. However, no direct morphoanatomical measurements of the ostiole aperture were obtained in this study. This interpretation should thus be regarded as a working hypothesis requiring direct morphoanatomical evaluation before any causal conclusion can be drawn. These findings suggest that breeding programs aimed at reducing ostiolar exposure or improving ostiolar integrity could help lower the risk of contamination. Likewise, agronomic practices that reduce ostiole-end splitting, including regulated deficit irrigation, may help reduce fungal ingress in susceptible cultivars [41].

The second major finding of this study relates to the contrasting ecology of *Aspergillus* sections *Flavi* and *Nigri*. Whereas section *Flavi* colonization was primarily determined by fig cultivar susceptibility, section *Nigri* colonization was strongly determined by fruit position, with ground-collected fruits consistently exhibiting higher colonization levels than canopy fruits across cultivars. Species within section *Nigri* are strongly associated with soil and saprotrophic environments [42], and ground fruits likely provide favorable microenvironmental conditions during drying and overripening. In contrast, the absence of a positional effect for section *Flavi* was likely due to the experimental inoculation design, which exposed all fruits to similar conidial loads regardless of their subsequent position. From a practical perspective, these findings suggest that minimizing the residence time of fruits on the soil surface may be particularly important for reducing the risk of OTA contamination.

The inoculation-method experiment also provided important methodological insight. Dry conidial inoculation produced substantially higher colonization than inoculation with aqueous suspensions. The mechanistic basis of this difference cannot be determined from the present data alone. The markedly lower colonization observed under aqueous suspension inoculation may reflect reduced inoculum adhesion to the hydrophobic fruit surface, premature conidial germination under high moisture conditions prior to host contact, or the physical loss of inoculum during application, as previously suggested by Doster and Michailides (1994) [43]. Regardless of the underlying mechanism, dry inoculation more closely approximates the mode of natural dispersal by wind currents and insect vectors, which deliver inoculum directly into the ostiolar canal, and these findings support its use in standardized susceptibility screening protocols for figs.

Our industrial surveillance dataset provided evidence consistent with the experimental findings. Importantly, these data were generated through the routine pre-commercialization screening program implemented by a local fig-processing cooperative. There, batches exceeding regulatory thresholds are systematically identified and removed before entering the commercial chain. Thus, the dataset reflects contamination levels prior to industrial exclusion procedures and should not be interpreted as representative of total orchard production or of consumer exposure levels. These data nonetheless highlight the importance of rigorous prescreening systems in maintaining food safety standards in the dried fig industry. Overall, ‘Calabacita’ exhibited consistently higher AF-related non-compliance than ‘Granito’, supporting the susceptibility patterns observed under controlled and field conditions. Considerable interannual variability was nevertheless observed, emphasizing the strong influence of seasonal climatic conditions on fungal colonization and mycotoxin accumulation [44]. Such variability highlights the limitations of single-season risk assessments and supports the need for long-term surveillance approaches in dried fig production systems. It should also be noted that detailed information on postharvest handling practices (including drying conditions, storage duration, sorting, and processing) was not consistently available for all batches in the surveillance dataset. The potential influence of these variables on final mycotoxin levels therefore remains an important area for future investigation.

The mycotoxin profile-based species attribution suggested that *Aspergillus* section *Nigri* accounted for a substantial proportion of OTA-related exceedances, particularly in ‘Granito’. Although this attribution is indicative rather than definitive, the pattern is ecologically consistent with the stronger soil association and competitive ability of *A. parasiticus* under warm and semi-arid pre-harvest conditions [44,45]. Consequently, management practices minimizing direct fruit–soil contact during drying, such as raised drying mats or off-ground drying systems, may be particularly important for reducing contamination by this highly toxigenic species. Furthermore, the relative contributions of the two *Aspergillus* sections varied across cultivars and years, suggesting that contamination dynamics are likely shaped by interactions among climate, orchard management, and fruit morphology.

The near absence of simultaneous AF and OTA exceedances within the same batches is consistent with the hypothesis that sections *Flavi* and *Nigri* occupy largely distinct ecological niches during fig drying and processing. This pattern may simplify risk stratification, although simultaneous monitoring of both toxin groups remains essential because each is associated with distinct fungal communities and management strategies.

Finally, the use of *A. flavus* for controlled inoculation was motivated not only by its epidemiological relevance but also by its potential use in biological control programs based on atoxigenic strains. Unlike *A. parasiticus*, whose known strains are consistently toxigenic, atoxigenic *A. flavus* strains can competitively exclude toxigenic populations from infection sites [22]. By demonstrating that fruit colonization largely depends on ostiolar access and cultivar susceptibility, this study provides mechanistic support for the future development of pre-harvest biocontrol strategies in European fig production systems. Similar approaches have already shown efficacy in other AF-susceptible crops such as maize [46], peanuts [21,47], and pistachios [20].

## 5. Conclusions

This study demonstrates marked differences in susceptibility to *Aspergillus* section *Flavi* among dried fig cultivars from the Jerte Valley. These differences were consistently observed in controlled inoculations, orchard assays, and, most importantly, in the systematic surveillance data from a local fig-processing cooperative, with cultivar comparisons based on the comparable period 2020–2023 (four harvest seasons). Overall, the results indicate that ‘Calabacita’ is considerably more susceptible to AF contamination than ‘Granito’, and the surveillance data are consistent with cultivar susceptibility being a major determinant of AF contamination risk in commercial batches. Ostiole-sealing experiments identified ostiolar accessibility as the principal determinant of infection, suggesting that morphological traits associated with the ostiole play a key role in fruit susceptibility. In addition, fruits in contact with the soil exhibited a particularly high risk of contamination by *Aspergillus* section *Nigri* species and OTA, highlighting the importance of minimizing fruit–soil contact during drying. The industrial dataset used in this study constitutes, to our knowledge, one of the most detailed longitudinal mycotoxin monitoring series reported for European dried figs. It is also the first such dataset evaluated under the framework of Commission Regulation (EU) 2023/915, which introduced a specific maximum level for OTA in dried figs. Surveillance data revealed repeated AF and OTA exceedances, with marked interannual variability. These results emphasize the importance of long-term monitoring programs and rigorous industrial prescreening systems prior to commercialization, such as those implemented by the industry to ensure that non-compliant batches are removed before entering the commercial chain. Together, these findings demonstrate that cultivar susceptibility, ostiolar accessibility, and fruit–soil contact are major determinants of mycotoxin contamination risk in dried figs. Based on these demonstrated relationships, cultivar selection, the reduction in soil–fruit contact during drying, and rigorous industrial prescreening are identified as priority areas for pre- and postharvest risk management.

## Data Availability

The original contributions presented in the study are included in the article/Supplementary Materials, further inquiries can be directed to the corresponding authors.

## Supplementary Materials

The following supporting information can be downloaded at https://www.mdpi.com/article/10.3390/foods15132289/s1, Figure S1: Commodity-specific breakdown of aflatoxins (AF) and ochratoxins (OTA) alerts in Spanish dried fruit and related products (2020–2025), by year. Each panel shows the distribution of alerts across food categories for AF (upper row) and OTA (lower row). Numbers in parentheses indicate the count of alerts per commodity. Data source: RASFF database (products of Spanish origin); Table S1: Number of non-compliant batches (%) exceeding EU maximum levels (Commission Regulation (EU) 2023/915) for aflatoxin B1 (AFB1 > 6 μg/kg), total aflatoxins (AFTA > 10 μg/kg), and ochratoxin A (OTA > 8 μg/kg) in commercial dried fig batches from the industry surveillance (2014–2023), by cultivar, year, and putative producing species.

## Abbreviations

The following abbreviations are used in this manuscript:

AF	Aflatoxins
AFPA	*Aspergillus flavus-parasiticus* agar
AFTA	Sum of total aflatoxins = AFB_1_ + AFB_2_ + AFG_1_ + AFG_2_
CICYTEX	Centro de Investigación Científica y Tecnológica de Extremadura
EPICON	Epidemiology and Control Lab of the University of Cordoba
OTA	Ochratoxin A
