# Supplementary material for: Susceptibility of Ficus carica L. Cultivars to Fruit Colonization by Aspergillus spp. and Its Relationship with Mycotoxin Contamination in Industrial Batches
Source: Foods. 2026 Jun 26;15(13):2289. doi: 10.3390/foods15132289 (PMC13360914; doi:10.3390/foods15132289)
Supplement: Supplementary file 1 [file foods-15-02289-s001.zip › foods-4372880-supplementary.pdf]

Table S1. Number of non-compliant batches (%) exceeding EU maximum levels (Commission Regulation (EU) 2023/915) for aflatoxin B_1 (AFB1 > 6 µg/kg), total aflatoxins (AFTA > 10 µg/kg), and ochratoxin A (OTA > 8 µg/kg) in commercial dried fig batches from the industry surveillance (2014–2023), by cultivar, year, and putative producing species.

			A. flavus		*A. parasiticus*		*Nigri*	TOTAL
**Cultivar**	**n**	**Year**	**AFB1 > 6**	**AFTA > 10**	**AFB1 > 6**	**AFTA > 10**	**OTA > 8**	**>Toxins limits**
**Granito**	11	2014	-	-	-	-	-	0 (0.0%) b
	54	2015	-	-	4 (7.4%) a	4 (7.4%) a	-	4 (7.4%) b
	38	2016	-	-	-	-	8 (21.1%) a	8 (21.1%) a
	39	2017	1 (2.6%) a	1 (2.6%) a	1 (2.6%) a	1 (2.6%) a	4 (10.3%) a	6 (15.4%) a
	9	2020	-	-	-	-	-	0 (0.0%) b
	47	2021	-	-	9 (19.1%) a	10 (21.3%) a	5 (10.6%) a	14 (29.8%) a
	33	2022	6 (18.2%) a	5 (15.2%) a	2 (6.1%) a	6 (18.2%) a	3 (9.1%) a	14 (42.4%) a
	25	2023	2 (8.0%) a	2 (8.0%) a	2 (8.0%) a	2 (8.0%) a	4 (16.0%) a	9 (36.0%) a
	**256**	**2014–2023**	**9 (3.5%) B**	**8 (3.1%) B**	**18 (7.0%) A**	**23 (9.0%) A**	**24 (9.4%) A**	**55 (21.5%) A**
**Calabacita**	4	2020	-	-	1 (25.0%) a	1 (25.0%) a	1 (25.0%)	2 (50.0%) a
	5	2021	-	-	-	-	-	0 (0.0%) a
	37	2022	11 (29.7%) a	10 (27.0%) a	1 (2.7%) a	1 (2.7%) b	-	12 (32.4%) a
	24	2023	-	-	5 (20.8%) a	6 (25.0%) a	-	6 (25.0%) a
	**70**	**2020–2023**	**11 (15.7%) A**	**10 (14.3%) A**	**7 (10.0%) A**	**8 (11.4%) A**	**1 (1.4%) B**	**20 (28.6%) A**

[a]Attribution by mycotoxin profile: batches exceeding OTA > 8 µg/kg were assigned to *Aspergillus* section *Nigri*. Among batches exceeding AFB1 > 6 µg/kg or AFTA > 10 µg/kg, those with detectable AFG_1 and/or AFG_2 were assigned to *A. parasiticus*, and those with AFB-only profiles to *A. flavus*. Batches exceeding both aflatoxin and OTA limits are reported in two columns (their aflatoxin attribution and Nigri-OTA) but counted only once in the TOTAL. A dash (–) indicates that no batches were assigned to the corresponding *Aspergillus* species in that year; '0 (0.0%)' indicates that batches were assessed but none exceeded the regulatory limit.

[b]Batches exceeding AFTA > 10 µg/kg with neither AFB nor AFG individually quantified were assigned to *A. flavus* in the absence of AFG.

Statistical comparisons. Lowercase letters indicate among-year differences within each cultivar and column, tested with the Zar multiple-comparison test for proportions (Zar, Biostatistical Analysis, §23.4; Tukey-type q statistic on the studentized range distribution, k groups, df = ∞, α = 0.05). Years sharing a letter do not differ significantly. Uppercase letters in the pooled rows compare cultivars over the full surveillance period (Granito 2014 – 2023, n = 256; Calabacita 2020 – 2023, n = 70) using Pearson's χ^2 with Yates' continuity correction; Fisher's exact test was applied when any expected frequency was below 5 (Cochran's rule). Cultivars sharing a letter do not differ significantly (α = 0.05).

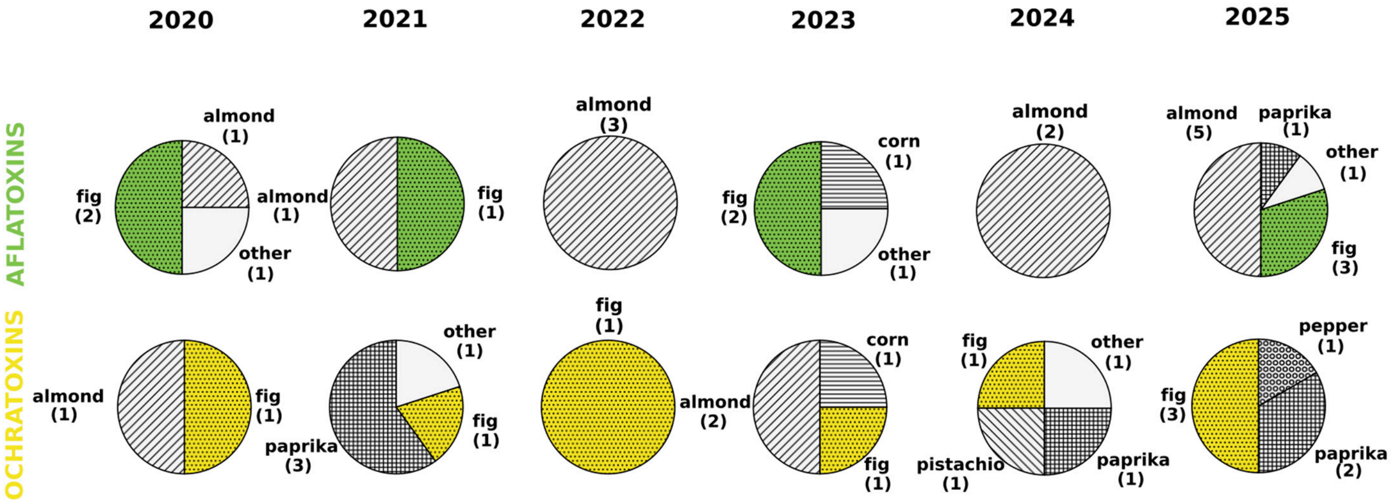

Figure S1. Commodity-specific breakdown of aflatoxins (AF) and ochratoxins (OTA) alerts in Spanish dried fruit and related products (2020-2025), by year. Each panel shows the distribution of alerts across food categories for AF (upper row) and OTA (lower row). Numbers in parentheses indicate the count of alerts per commodity. Data source: RASFF database (products of Spanish origin).
